# Supplementary material for: Effects of Live Music Therapy on Autonomic Stability in Preterm Infants: A Cluster-Randomized Controlled Trial
Source: Children (Basel). 2021 Nov 22;8(11):1077. doi: 10.3390/children8111077 (PMC8618386; doi:10.3390/children8111077)
Supplement: Supplementary file 1 [file children-08-01077-s001.zip › supplementary tables_version for publication.pdf]

**Supplementary material**

*Table S1: Change in HF power within the second session, mothers and fathers separately*

| **Mothers** (102 observations) | **B (SE)** | ***P*-Value** |
|---|---|---|
| Intercept [a] | 8.47 (0.58) | <0.001*** |
| Session part: last | 4.52 (0.82) | <0.001*** |
| Treatment: MT+SSC | 1.13 (0.81) | 0.201 |
| Session part: last X treatment: MT+SSC | 8.03 (1.14) | <0.001*** |
| **Fathers** (22 observations) | | |
| Intercept | 7.41 (2.86) | 0.022* |
| Session part: last | 1.42 (3.09) | 0.653 |
| Treatment: MT+SSC | 3.35 (3.07) | 0.326 |
| Session part: last X treatment: MT+SSC | -0.57 (3.31) | 0.867 |

HF, high frequency; MT, music therapy; SSC, skin-to-skin care

[a] Intercept is the predicted value for the sample in the first part of the 2nd SSC session.

B, beta coefficient; SE, standard error.

*Table S2: Primary outcome: covariate examination*

| **HF: Change within session + Covariates** (no. of observations: 124**)** | **B (SE)** | ***P*-value** |
|---|---|---|
| Intercept | 16.44 (12.08) | 0.177 |
| Session part: last | 4.52 (0.96) | <0.001*** |
| Parent: Fathers | -3.61 (1.90) | 0.061 |
| Infant gender: Female | 0.24 (1.03) | 0.821 |
| Gestational Age in weeks | -0.20 (0.35) | 0.556 |
| Postnatal age in days at T1 | -0.05 (0.05) | 0.404 |
| Birthweight | -0.00 (0.00) | 0.800 |
| Neonatal medical index grade | 0.01 (1.16) | 0.992 |
| Treatment: MT+SSC | -10.55 (16.51) | 0.524 |
| Session part: last X treatment: MT+SSC | 5.05 (1.29) | <0.001*** |
| Parent: Fathers X treatment: MT+SSC | -1.21 (2.18) | 0.581 |
| Infant gender: Female X treatment: MT+SSC | -0.36 (1.41) | 0.807 |
| Gestational Age in weeks X treatment: MT+SSC | 0.34 (0.49) | 0.486 |
| Postnatal age in days at T1 X treatment: MT+SSC | 0.10 (0.07) | 0.188 |
| Birthweight X treatment: MT+SSC | 0.00 (0.00) | 0.891 |
| Neonatal medical index grade X treatment: MT+SSC | -0.07 (1.41) | 0.962 |

HF, high frequency; MT, music therapy; SSC, skin-to-skin care

[a] Intercept is the predicted value for mothers in the first part of the 2nd SSC session.

B, beta coefficient; SE, standard error.

*Table S3: Observed values of HRV parameters in all 3 sessions*

| Variable | MT+SSC | SSC | *P*-value[a] | Effect Size |
|---|---|---|---|---|
| | ms2/Hz (SD) | ms2/Hz (SD) | | d (95% CI) |
| Mean HF T1 | 12.34(2.50) | 12.15(2.07) | .739 | .081(-.402, .565) |
| Mean HF T1M | 12.20 (2.64) | 12.09(2.34) | .874 | .040(-.463, .544) |
| Mean HF T1F | 12.36(2.71) | 12.30(2.71) | .963 | .023(-.930, .975) |
| Mean LF T1 | 16.94(3.03) | 17.74(5.64) | .485 | -.180(-.663, .305) |
| Mean LF T1M | 17.49(2.80) | 18.72(5.52) | .292 | -.288(-.793, .219) |
| Mean LF T1F | 12.61(1.99) | 12.41(2.63) | .866 | .086(-.896, 1.065) |
| Mean LF/HF ratio T1 | 1.49(0.48) | 1.53(0.61) | .799 | -.065(-.552, .423) |
| Mean LF/HF ratio T1M | 1.54(0.48) | 1.64(0.62) | .501 | -.181(-.689, .330) |
| Mean LF/HF ratio T1F | 1.14(0.27) | 1.00(0.24) | .302 | .537(-.472, 1.527) |
| Mean HF T2 | 12.74(3.22) | 13.02(2.67) | .708 | -.095(-.600, .411) |
| Mean HF T2M | 12.85(3.65) | 13.10(2.77) | .771 | -.077(-.601, .447) |
| Mean HF T2F | 12.81(2.30) | 13.90(1.500) | .386 | -.503(-1.815, .834) |
| Mean LF T2 | 12.63(2.45) | 15.20(3.20) | .001*** | -.916(-1.443, -.381) |
| Mean LF T2M | 13.02(2.33) | 15.52(3.26) | .002*** | -.892(-1.434, -.342) |
| Mean LF T2F | 11.59(3.25) | 11.27(2.31) | .858 | .105(-1.205, 1.410) |
| Mean LF/HF ratio T2 | 1.11(0.38) | 1.26(0.40) | .145 | -.384(-.892, .128) |
| Mean LF/HF ratio T2M | 1.16(0.41) | 1.28(0.40) | .274 | -.293(-.814, .231) |
| Mean LF/HF ratio T2F | 0.92(0.29) | 0.73(0.15) | .189 | .713(-.646, 2.039) |
| Mean HF T3 | 13.54(2.39) | 12.48(2.24) | .109 | .458(-.106, 1.017) |
| Mean HF T3M | 13.73(2.52) | 12.21(2.22) | .038* | .632(.025, 1.231) |
| Mean HF T3F | 12.62(2.40) | 14.73(1.19) | .149 | -1.016(-2.519, .557) |
| Mean LF T3 | 17.73(2.99) | 22.06(2.86) | .001*** | -1.108(-1.699, -.508) |
| Mean LF T3M | 18.52(2.24) | 23.33(4.47) | .000*** | -1.414(-2.067, -.748) |
| Mean LF T3F | 13.14(2.26) | 13.00(4.59) | .964 | .043(-1.390, 1.473) |
| Mean LF/HF ratio T3 | 1.37(0.27) | 1.91(0.53) | .000*** | -1.336(-1.944, -.716) |
| Mean LF/HF ratio T3M | 1.41(0.27) | 2.06(0.49) | .000*** | -1.697(-2.378, -1.002) |
| Mean LF/HF ratio T3F | 1.14(0.32) | 0.87(0.30) | .288 | .865(-.675, 2.343) |
| Mean HF all sessions | 12.82(1.77) | 12.60(1.26) | .553 | .141(-.337, .618) |
| Mean HF all sessions, M | 12.76(2.39) | 12.50(1.39) | .565 | .138(-.353, .627) |
| Mean HF all sessions, F | 12.71(2.15) | 12.98(2.37) | .797 | -.117(-.981, .749) |
| Mean LF all sessions | 15.61(1.71) | 17.63(3.12) | .002** | -.826(-1.321, -.326) |
| Mean LF all sessions, M | 16.14(1.64) | 18.47(2.84) | .000*** | -1.032(-1.550, -.507) |
| Mean LF all sessions, F | 12.17(2.06) | 11.99(2.21) | .859 | .084(-.813, .977) |

| | | | | |
|---|---|---|---|---|
| Mean LF/HF ratio all sessions | 1.31(0.28) | 1.50(0.36) | .025 | -.574(-1.059, -.085) |
| Mean LF/HF ratio all sessions, M | 1.38(0.30) | 1.59(0.33) | .010* | -.670(-1.170, -.165) |
| Mean LF/HF ratio all sessions, F | 1.03(0.20) | 0.90(0.12) | .096 | .726(-.208, 1.642) |
| HF change T2 | 9.80(6.89) | 4.43(2.21) | .000*** | .998(.438, 1.549) |
| HF change T2M | 12.55(5.35) | 4.52(2.21) | .000*** | 1.946(1.269, 2.610) |
| HF change T2F | 0.85(4.47) | 1.10(N/A) | .960[1] | -.055(-2.119, 2.103) |
| LF change T2 | -5.78(3.16) | -8.47(3.67) | .005** | .791(.245, 1.332) |
| LF change T2M | -6.37(3.29) | -8.65(4.12) | .035* | .612(.047, 1.172) |
| LF change T2F | -4.48(2.99) | -6.17(2.77) | .425 | .572(-.771, 1.889) |
| LF/HF ratio change T2 | -1.18(0.61) | -1.62(0.73) | .020* | .660(.119, 1.194) |
| LF/HF ratio change T2M | -1.37(0.69) | -1.71(0.73) | .096 | .476(-.084, 1.030) |
| LF/HF ratio change T2F | -0.65(0.53) | -0.43(0.58) | .599 | -.409(-1.718, .919) |

MT, music therapy; SSC, skin-to-skin care; HF, high frequency; LF, low frequency; T1, first session, T2, second session, T3, third session; M, mothers, F, fathers.

[a] *P*-values are from t-tests not assuming equal variances except where indicated. [1] n=1; p-value only given for equal variance assumed. * $p < 0.05$; ** $p < 0.01$; *** $p < 0.001$.

*Table S4: Observed values of parental scores on the Maternal Postnatal Attachment Scale and State Trait Anxiety Inventory*

| **Variable** | **MT+SSC** | **SSC** | ***P*-Value[a]** | **Effect Size** |
|---|---|---|---|---|
| Maternal Postnatal Attachment Scale | M(SD) | M(SD) | | d (95% CI) |
| T1 | 77.99(8.33) | 80.95(6.10) | .102 | -.399(-.887, .092) |
| T1. M | 78.35(8.72) | 81.25(5.48) | .117 | -.390(-.898, .122) |
| T1. F | 76.00(6.78) | 81.28(7.43) | .119 | -.745(-1.649, .178) |
| T2 | 75.65(6.77) | 81.67(5.77) | .031* | -.977(-1.795, -.142) |
| T2. M | 76.71(7.55) | 81.32(5.80) | .133 | -.717(-1.542, .123) |
| T2. F | 76.07(4.08) | 90.00 | .055[1] | -3.410(-6.716, .066) |
| T3 | 79.17(4.49) | 79.74(8.08) | .767 | -.091(-.645, .464) |
| T3. M | 79.91(4.76) | 80.81(6.32) | .598 | -.165(-.753, .425) |
| T3. F | 75.28(3.79) | 72.10(14.45) | .741 | .381(-1.032, 1.767) |
| State Trait Anxiety Inventory (STAI) | | | | |
| T1 Pre | 34.92(11.62) | 32.17(7.58) | .248 | .275(-.210, .757) |
| T1.M Pre | 35.74(13.62) | 32.00(8.12) | .184 | .324(-.183, .828) |
| T1.F Pre | 33.83(10.68) | 34.11(10.31) | .953 | -.026(-.890, .838) |
| T1 Post | 26.67(7.68) | 26.63(5.86) | .980 | .006(-.475, .488) |
| T1.M Post | 27.40(8.92) | 26.81(7.10) | .775 | .072(-.431, .573) |
| T1.F Post | 25.67(4.29) | 27.67(8.72) | .539 | -.307(-1.173, .566) |
| T2 Pre | 35.12(10.64) | 29.13(6.95) | .014* | .659(.122, 1.190) |
| T2.M Pre | 37.00(12.11) | 29.36(8.12) | .010* | .735(.169, 1.295) |
| T2.F Pre | 31.00(5.00) | 31.20(10.35) | .970 | -.026(-1.173, 1.122) |
| T2 Post | 27.08(6.73) | 25.85(6.80) | .496 | .182(-.340, .702) |
| T2.M Post | 27.92(7.82) | 25.64(7.53) | .288 | .298(-.251, .843) |
| T2.F Post | 26.57(5.06) | 27.80(5.07) | .689 | -.243(-1.389, .916) |
| T3 Pre | 28.90(6.19) | 28.39(8.11) | .807 | .072(-.483, .626) |
| T3.M Pre | 27.83(6.08) | 27.10(6.10) | .693 | .120(-.474, .714) |
| T3.F Pre | 34.50(6.83) | 35.00(16.70) | .964 | -.047(-1.431, 1.341) |
| T3 Post | 23.59(3.96) | 23.39 | .853 | .052(-.502, .606) |
| T3.M Post | 23.29(4.04) | 22.85(3.17) | .686 | .120(-.474, .714) |
| T3.F Post | 25.67(3.67) | 26.00(6.00) | .936 | -.075(-1.459, 1.314) |
| Change in STAI scores, pre to post test | | | | |
| T1 | -8.24(7.51) | -5.53(4.01) | .064 | -.437(-.923, .052) |
| T1.M | -8.34(7.80) | -5.18(4.22) | .050* | -.476(-.983, .035) |
| T1.F | -8.17(8.55) | -6.44(4.13) | .550 | -.245(-1.109, .626) |
| T2 | -8.03(7.59) | -3.28(3.76) | .004** | -.781(-1.318, -.238) |
| T2. M | -9.07(7.90) | -3.72(4.56) | .004** | -.822(-1.385, -.251) |
| T2. F | -4.43(3.95) | -3.40(5.90) | .745 | -.213(-1.359, .943) |

| | | | | |
|---|---|---|---|---|
| T3 | -5.31(4.42) | -5.00(5.54) | .830 | -.063(-.617, .492) |
| T3. M | -4.54(4.19) | -4.25(4.40) | .824 | -.068(-.661, .526) |
| T3. F | -8.83 | -9.00(10.82) | .982 | .021(-1.365, 1.407) |

MT, music therapy; SSC, skin-to-skin care; T1, first session, T2, second session, T3, third session; M, mothers, F, fathers.

[a]*P*-values are from t-tests not assuming equal variances except where indicated. [1] n=1; p-value only given for equal variance assumed. * p < 0.05; ** p < 0.01; *** p< 0.001.
