# Supplementary figures and images for: Effects of Live Music Therapy on Autonomic Stability in Preterm Infants: A Cluster-Randomized Controlled Trial
Source: Children (Basel). 2021 Nov 22;8(11):1077. doi: 10.3390/children8111077 (PMC8618386; doi:10.3390/children8111077)

## CONSORT 2010 Flow Diagram

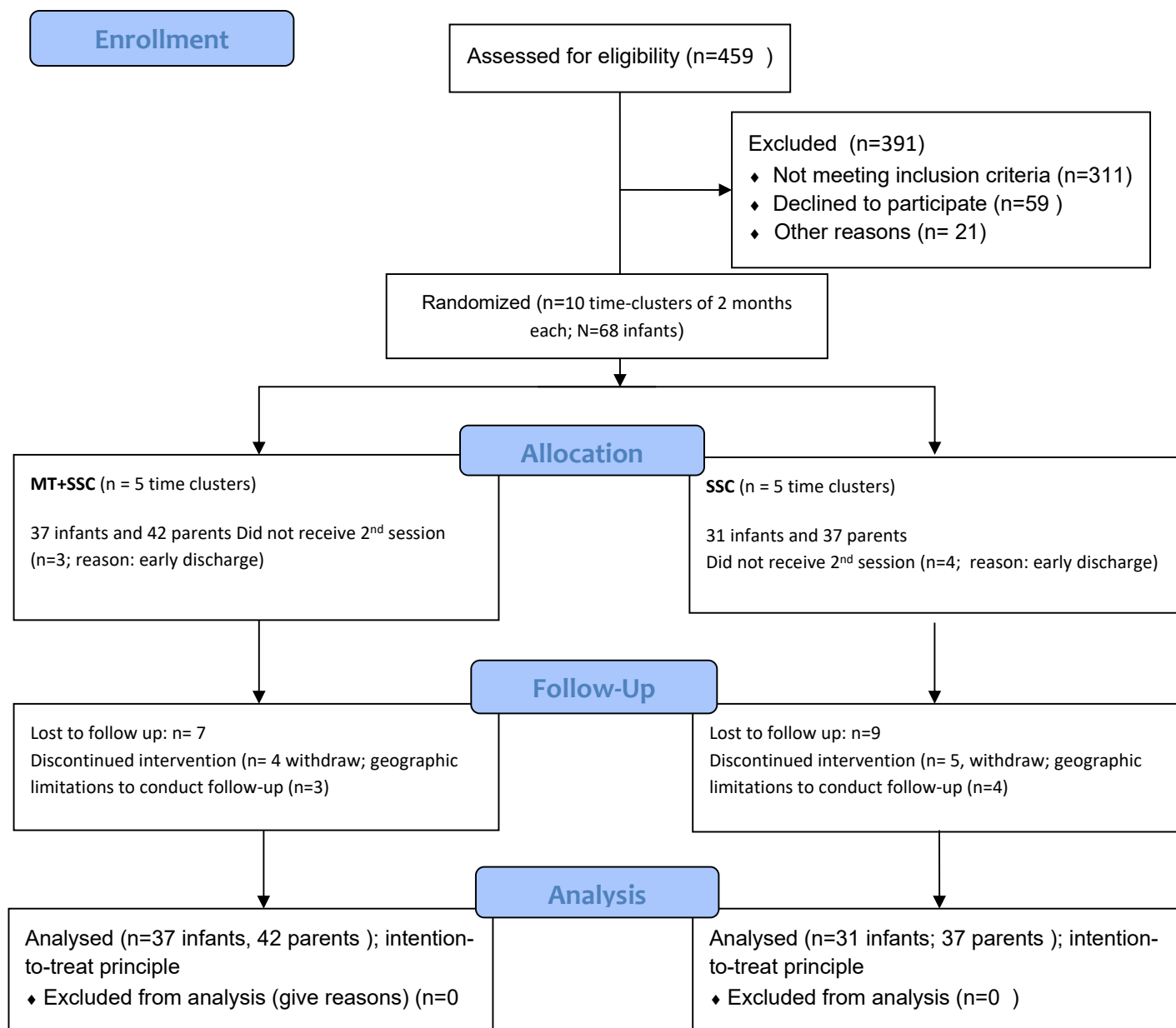

Supplement: Supplementary file 1 [file children-08-01077-s001.zip › consort-2010-flow-diagram_children_effects of MT and SSC.pdf]
